# Global variability in colorectal cancer screening guidelines: panel composition and recommendations

**DOI:** 10.1093/jcag/gwag024

**Published:** 2026-07-01

**Authors:** Koki Nakajima, Hiroaki Saito, Yoshitaka Nishikawa

**Affiliations:** Japanese Red Cross Wakayama Medical Center, Wakayama City, Wakayama 640-8558, Japan; Department of Internal Medicine, Soma Central Hospital, Soma City, Fukushima 976-0016, Japan; Department of Radiation Health Management, Fukushima Medical University School of Medicine, Fukushima City, Fukushima 960-1295, Japan; Department of Health Informatics, Kyoto University School of Public Health, Kyoto City, Kyoto 606-8501, Japan; Department of Health Informatics, Kyoto University School of Public Health, Kyoto City, Kyoto 606-8501, Japan; Takemi Program in International Health, Harvard T. H. Chan School of Public Health, Boston, MA 02115, United States

**Keywords:** colorectal neoplasms, screening, guideline, conflict of interest, international comparison

## Abstract

**Objective:**

Colorectal cancer (CRC) is a leading cause of cancer-related mortality worldwide. Although many countries have developed CRC screening guidelines, how CRC screening guidelines are developed across countries remains unclear. This study examined guideline-development funding, stakeholders, and their conflicts of interest (COI), and compared CRC screening recommendations across Group of Seven (G7) countries.

**Methods:**

We reviewed the most recent CRC screening guidelines issued by official research organizations or governmental bodies in G7 countries. We extracted information on responsible organizations, panel composition, recommendations, target ages, screening tests and intervals, supporting evidence, funding, and COI.

**Results:**

While CRC screening recommendations showed some similarities across G7 countries, some differences were observed in starting age, screening modality, and implementation structure. Differences were also identified in funding, COI reporting and management, and stakeholder composition, including limited involvement of patient and public representatives.

**Conclusions:**

Colorectal cancer screening guidelines among G7 countries varied despite shared international evidence. These differences likely reflect country-specific factors, including population characteristics, screening participation, health system capacity, and cultural context. Given that guideline development involves diverse stakeholders, ensuring transparency in the guideline-development process is essential.

Colorectal cancer (CRC) is the third most common cancer worldwide, accounting for approximately 10% of all cancer cases, and is the second leading cause of cancer-related deaths globally.^1^ While screening programs reduce CRC incidence and mortality,^2^ their effect on overall life expectancy remains a subject of ongoing debate, as previous research has raised debate over whether such programs extend overall life expectancy.^3^ When developing clinical practice guidelines, it is crucial not only to consider the strength and type of evidence, but also to examine how each recommendation was made. However, this process across different countries remains little understood. This study aimed to examine guideline-development stakeholders and compare recommendations in Group of Seven (G7) nations, which have relatively high CRC incidence and established screening guidelines.

We reviewed the most recent CRC screening guidelines issued by official research organizations or governmental bodies in each G7 country (Canada, France, Germany, Italy, Japan, the United Kingdom (UK), and the United States (US)). For the evaluation items used in this study, in addition to guideline recommendations, we referred to selected AGREE II, especially domains 2 and 6, which were considered most relevant to the purpose of this analysis. Specifically, we collected information on the organizations responsible for guideline development, funding of guideline development, the composition of their development panels, conflicts of interest (COI) of panel members, recommendations (recommended tests and screening intervals, target age ranges), and the evidence cited to support each recommendation. Regarding the composition of the guideline development committees, panel members were categorized into 6 groups based on their roles: Clinicians, Public Health Experts, Methodologists, Non-clinical Advisors, Government Officers, and Patient and Public Representatives (PPR).

In addition, to reduce subjectivity in the assessment process, the funding and COI items within AGREE II domain 6 were independently evaluated by the author and a co-authors in accordance with the AGREE II manual (score range, 1-7; 1 = strongly disagree, 7 = strongly agree), and average scores were calculated.

Further details on data collection and guideline access are provided in the Appendix S1.

Table 1 summarizes the CRC screening guideline characteristics across G7 nations. Regarding the funding of the organizations responsible for guideline development and the COI management of panel members, these organizations are generally structured to operate independently from their funders and to prevent inappropriate influence on committee members (Appendix S2). However, based on our evaluation using the AGREE II instrument (domain 6, items 22 and 23), the extent to which this independence is explicitly described varies across guidelines; some provide detailed statements in the guideline affirming that funders did not influence the recommendations and that COIs were strictly managed, whereas others offer only limited or no information regarding funding and COI in the guideline.

**Table 1:** Summary of the CRC screening guideline characteristics across G7 countries.

Nation	Organization	Panel member composition [*n* (%)]						Guideline recommendations		
		Clinician	Public health expert	Methodologist	Non-clinical advisor	Government officer	PPR	Test	Interval [year]	Target age [years old]
**Canada (2016)**	CTFPHC	7 (64%)	1 (9%)	0	0	3 (27%)	0	FOBT	2	50-74
								FS	10	
**France (2013)**	Haute Autorité de Santé	2 (9%)	18 (81%)	0	1 (5%)	1 (5%)	0	FIT	2	50-74
**Germany (2026)**	AWMF	4 (33%)	1 (8%)	4^a^ (33%)	0	0	3 (25%)	Colonoscopy	10	Over 50
								FS	5	
								FOBT	1-2	
**Italy (2024)**	AIOM	26 (84%)	0	5^a^ (16%)	0	0	0	FOBT	2	50-69
								FS	Once-in-a-lifetime	55-64
**Japan (2024)**	National Cancer Center	3 (38%)	4 (50%)	0	0	0	1 (13%)	FIT	1-2	40-74
**UK (2018)**	National Screening Committee	4 (29%)	7 (50%)	1^a^ (7%)	0	0	2 (14%)	FIT	2	51-65 (Current Capacity)
										50-74 (Ideal Capacity)
**US (2021)**	USPSTF	14 (78%)	4 (22%)	0	0	0	0	Options^b^	Up to test	45-75 and 76-85^b^

a In some countries, teams providing methodological support were involved in the development of guidelines. Specifically, 4 individuals in Germany, 5 in Italy, and 6 in the United Kingdom participated in addition to the committee members, supporting the guideline development process.

b The United States offers the following screening options: high-sensitivity guaiac fecal occult blood test (HSgFOBT) or fecal immunochemical test (FIT) annually; stool DNA-FIT every 1-3 years; computed tomography colonography every 5 years; flexible sigmoidoscopy every 5 years; flexible sigmoidoscopy every 10 years combined with annual FIT; or colonoscopy every 10 years. Specifically for adults aged 76-85 years, the United States recommends that clinicians selectively offer colorectal cancer screening, based on individual patient health status, prior screening history, and personal preferences, given the small net benefit of screening in this age group.

Abbreviations: AIOM = Italian Association of Medical Oncology (Associazione Italiana di Oncologia Medica); AWMF = Association of the Scientific Medical Societies in Germany (Arbeitsgemeinschaft der Wissenschaftlichen Medizinischen Fachgesellschaften e. V.); CTFPHC = Canadian Task Force on Preventive Health Care; FIT = fecal immunochemical test; FOBT = fecal occult blood test; FS = flexible sigmoidoscopy; PPR = patient and public representative; USPSTF = United States Preventive Services Taskforce.

Regarding the composition of panel members, while clinicians and public health experts made up the majority in all countries, the proportion between these 2 groups, as well as the inclusion of members from other categories such as patient or public representatives (PPRs), varied across nations. Despite the recognized importance of PPR in trustworthy guideline development,^4^ their inclusion remained limited. As for the target age, most guidelines recommended the early 50 s through the mid-70s, while Japan lowered at 40 and the United States at 45. Japan’s earlier start age aligned with existing nationwide screening programs, while the U.S. guidelines recommended starting screening at 45 years old based on a study that showed that lowering the age from 50 to 45 resulted in 22-27 additional life-years gained per 1000 individuals, supporting earlier screening.^5^ The UK adjusted the target age range for colorectal cancer screening based on cost-effectiveness analyses, with the aim of optimizing resource allocation. The actual age range, however, was constrained by colonoscopy capacity for individuals with positive screening results. Under current capacity, the range was 51-65 years, whereas under ideal capacity, it would be extended to 50-74 years. In terms of screening tests, most G7 guidelines recommended the Fecal Immunochemical Test (FIT) or the Fecal Occult Blood Test (FOBT) as the primary screening, while Germany and the United States recommended multiple testing options. According to their guidelines, both countries proposed multiple methods because each screening test has its own advantages and disadvantages. In the case of Germany, the guideline emphasized the result of the NordICC trial, which demonstrated that colonoscopy reduced both the incidence and mortality of colorectal cancer.^6^ In line with this evidence, colonoscopy is currently implemented as the first-line screening method in the German national program.^7^ By contrast, colorectal cancer screening in the United States is conducted in an opportunistic manner within primary care rather than through a centrally organized national program. This distinction highlighted a structural difference in implementation, even though both countries recommended multiple testing strategies. For the interval, countries recommending FIT or FOBT typically recommended screening every 2 years, whereas those recommending sigmoidoscopy or colonoscopy generally recommended screening every 10 years.

Variation also existed within countries. In Canada, while most provinces recommended FIT every 2 years for average-risk individuals aged 50-74 years, some provinces used shorter screening intervals, and 1 province lowered the starting age to 45 years.^8^ Similarly, although FIT-based screening has been implemented nationwide in Japan, some municipalities have introduced colonoscopy-based screening programs for selected populations.^9^

CRC screening guidelines among G7 nations showed variability in their recommended practices, despite the availability of numerous global studies, including randomized controlled trials, on colorectal cancer prevention. This is partly because the development and implementation of screening guidelines cannot rely solely on international evidence; they must also incorporate country-specific factors such as population demographics, participation rates, health system capacity, and cultural context. Addressing these complexities requires contributions from a broad range of stakeholders, ensuring that panels are composed not only of clinical and methodological experts but also of governmental representatives, public health specialists, and patient and public representatives. Moreover, because diverse stakeholders participate in guideline development, the deliberation must be explicitly documented and publicly accountable. The documentation should specify the evidence considered, the value judgments applied, and the capacity constraints considered, so that decisions are legitimate and comparable across settings. Accordingly, ensuring transparency in the guideline development process is essential.

## Supplementary Material

gwag024_Supplementary_Data

## Data Availability

All guidelines used in this study are publicly available online. The data extracted and analyzed in this study are fully presented in the manuscript or the Appendix.

## References

[1] World Health Organization. Geneva: World Health Organization. Accessed April 5, 2025. https://www.who.int/news-room/fact-sheets/detail/colorectal-cancer

[2] Lieberman D , Ladabaum U, Brill JV, et al Reducing the burden of colorectal cancer: AGA position statements. Gastroenterology. 2022;163:520-526. 10.1053/j.gastro.2022.05.011 35715380

[3] Bretthauer M , Wieszczy P, Løberg M, et al Estimated lifetime gained with cancer screening tests: a meta-analysis of randomized clinical trials. JAMA Intern Med. 2023;183:1196-1203. 10.1001/jamainternmed.2023.3798 PMC10463170 37639247

[4] Armstrong MJ , Mullins CD, Gronseth GS, Gagliardi AR. Impact of patient involvement on clinical practice guideline development: a parallel group study. Implementation Sci. 2018;13:55. 10.1186/s13012-018-0745-6 PMC5902835 29661195

[5] Knudsen AB , Rutter CM, Peterse EFP, et al Colorectal Cancer Screening: An Updated Decision Analysis for the U.S. Preventive Services Task Force. Agency for Healthcare Research and Quality (US); 2021 (U.S. Preventive Services Task Force Evidence Syntheses, formerly Systematic Evidence Reviews). Accessed April 5, 2025. http://www.ncbi.nlm.nih.gov/books/NBK570833/

[6] Bretthauer M , Løberg M, Wieszczy P, et al NordICC Study Group. Effect of colonoscopy screening on risks of colorectal cancer and related death. N Engl J Med. 2022;387:1547-1556. 10.1056/NEJMoa2208375 36214590

[7] Gemeinsamer Bundesausschuss (G-BA). Berlin: Gemeinsamer Bundesausschuss. Accessed April 5, 2025. https://www.g-ba.de/themen/methodenbewertung/erwachsene/krebsfrueherkennung/darmkrebs-screening/

[8] Canadian Cancer Society. Toronto: Canadian Cancer Society. Accessed May 30, 2025. https://cancer.ca/en/cancer-information/find-cancer-early/get-screened-for-colorectal-cancer/how-do-i-find-a-colorectal-cancer-screening-program

[9] Kumamoto City. Kumamoto: Kumamoto City. Accessed May 30, 2025. https://www.city.kumamoto.jp/kiji00364997/index.html

