# Supplementary material for: Global variability in colorectal cancer screening guidelines: panel composition and recommendations
Source: J Can Assoc Gastroenterol. 2026 Jul 1;9(5):271–4. doi: 10.1093/jcag/gwag024 (PMC13639361; doi:10.1093/jcag/gwag024)
Supplement: Supplementary file 1 [file gwag024_supplementary_data.zip › Appendix.docx]

**Appendix** **1.** Details on data collection and guideline access

1. Access to Colorectal Cancer Screening Guidelines

We obtained these guidelines directly from each organization’s website. No formal database search was conducted, and no exclusion criteria were applied beyond selecting the latest available version. In cases where guidelines were written in languages other than English, we used DeepL (DeepL SE, Germany) for translation. The guidelines for each country analyzed in this study are as follows (as of April 5, 2026, using the most recent versions available):

Canada: Canadian Task Force on Preventive Health Care (CTFPHC), 2016 ^[1]^
France: French National Authority for Health (Haute Autorité de Santé, HAS), 2013 ^[2]^*
Germany: Association of the Scientific Medical Societies in Germany (Arbeitsgemeinschaft der Wissenschaftlichen Medizinischen Fachgesellschaften e.V., AWMF), 2026 ^[3]^
Italy: Italian Association of Medical Oncology (Associazione Italiana di Oncologia Medica, AIOM), 2024 ^[4]^
Japan: National Cancer Center (NCC), 2024 ^[5]^
United Kingdom: Sophie Whyte et al., 2018 ^[6]^**
United States: US Preventive Services Task Force (USPSTF), 2021 ^[7]^

2. Data Collection
We extracted and compared the following four factors from each guideline:

2-1. Organization
The organization responsible for guideline creation.

2-2. Funding

For each guideline, we reviewed the sources of funding for the guideline development process and for the organizations responsible for producing the guideline. Funding information was extracted primarily from sections within the guideline documents. When such descriptions were absent or insufficient, we researched the official websites and publicly available reports of the corresponding organizations. The extracted information was then independently evaluated by the author and two co-authors with the AGREE II instrument (score range, 1–7; 1 = strongly disagree, 7 = strongly agree), and the average scores were calculated.

2-3 Conflict of interest
Information on conflicts of interest (COI) among guideline development panel members was obtained from the guideline documents and, when necessary, from COI policies, disclosure forms, or related statements available on the organizations’ official websites. The extracted information was then independently evaluated by the author and two co-authors with the AGREE II instrument (score range, 1–7; 1 = strongly disagree, 7 = strongly agree), and the average scores were calculated.

2-4. Panel Members
The composition of the guideline development committee. Panel members were categorized into six groups based on their roles:

Clinicians: Healthcare professionals who provide direct patient care, such as physicians, nurses, pharmacists, and physical therapists.
Public Health Experts: Specialists involved in public health practice or policy, including public health physicians, epidemiologists, health administrators, ethicists, and health economists.
Methodologists: Experts in systematic reviews, evidence grading, and guideline methodology.
Non-clinical Advisors: Professionals from non-medical backgrounds such as law.
Government Officers: Personnel affiliated with national organizations responsible for healthcare administration and policy implementation.
Patient and Public Representatives (PPR): Advocates from patient organizations or the public.

This classification of roles was determined through discussions among the co-authors.

To categorize each member into one of the six groups, we first referred to the affiliations listed in the guideline documents. If affiliations were not provided or were insufficient to determine the member’s role, we consulted the affiliations listed in their publications on PubMed around the time the guideline was developed. When classification remained unclear, we referred to institutional websites and made decisions through discussions among the co-authors. In cases where a member held dual affiliations—such as departments of both internal medicine and public health—we classified the individual as a clinician, prioritizing their clinical role.

2-5. Recommendation
Recommended screening tests, specified intervals, and target ages for initiation and cessation.

2-6. Evidence
The primary studies or data cited to support each recommendation.

* For France, since the guideline did not list its authors, we referred to the official website of the Haute Autorité de Santé and summarized the members of the committee at that time. ^[8]^

** The UK National Screening Committee (NSC), which advises on national cancer screening policy, commissioned the School of Health and Related Research (ScHARR) at the University of Sheffield to conduct the modelling study of bowel cancer screening strategies. Regarding the panel members, we analyzed those of the UK National Screening Committee. However, as the composition of the panel members at the time the guideline was developed in 2018 was not available, our analysis was based on the members listed as of Apr 5, 2026.

| **Nation** | **Organization** | **Funding**  **[1–7, mean (n=3)]** | **COI**  **[1–7, mean (n=3)]** |
| --- | --- | --- | --- |
| **Canada (2016)** | CTFPHC | 7 | 6 |
| **France (2013)** | Haute Autorité de Santé | 1 | 1 |
| **Germany (2026)** | AWMF | 6.3 | 6.7 |
| **Italy (2024)** | AIOM | 1 | 6.3 |
| **Japan (2024)** | National Cancer Center | 6.3 | 6 |
| **UK (2018)** * | National Screening Committee | 4.7 | 1 |
| **US (2021)** | USPSTF | 7 | 6 |

**Appendix 2.** AGREE II–based assessment of funding and conflict of interest (COI) in CRC screening guidelines across G7 countries

Abbreviations: AIOM, Italian Association of Medical Oncology (Associazione Italiana di Oncologia Medica); AWMF, Association of the Scientific Medical Societies in Germany (Arbeitsgemeinschaft der Wissenschaftlichen Medizinischen Fachgesellschaften e.V.); CTFPHC, Canadian Task Force on Preventive Health Care; USPSTF, United States Preventive Services Taskforce.

**Reference**

1. Canadian Task Force on Preventive Health Care. Recommendations on screening for colorectal cancer in primary care. CMAJ. 2016 Mar;188(5):340–8. doi:10.1503/cmaj.151125 PubMed PMID: 26903355; PubMed Central PMCID: PMC4786388.
2. Haute Autorité de Santé. Saint-Denis La Plaine: Haute Autorité de Santé; 2013. [cited 2026 Apr 5]. Available from: <https://www.has-sante.fr/upload/docs/application/pdf/2013-07/referentieleps_format2clic_kc_colon-vfinale_2013-07-24_16-05-56_103.pdf>
3. Arbeitsgemeinschaft der Wissenschaftlichen Medizinischen Fachgesellschaften (AWMF). Berlin: Arbeitsgemeinschaft der Wissenschaftlichen Medizinischen Fachgesellschaften; 2026 Jun. [cited 2026 Apr 5]. Available from: https://www.leitlinienprogramm-onkologie.de/fileadmin/user_upload/Downloads/Leitlinien/Kolorektales_Karzinom/Version_3/LL_KRK_Langversion_3.1.pdf
4. Associazione Italiana di Oncologia Medica (AIOM). Milan: Associazione Italiana di Oncologia Medica; 2024. [cited 2026 Apr 5]. Available from: <https://www.iss.it/documents/20126/8403839/LG+279+Retto+e+Ano_agg2025.pdf/6b78cb84-7e6b-2fef-ce77-2dad8f64760b?t=1742888997568>
5. National Cancer Center, Japan. Tokyo: National Cancer Center; 2024 Jul 18. [cited 2026 Apr 5]. Available from: <https://canscreen.ncc.go.jp/guideline/colon_guideline2024.pdf>
6. Whyte S, et al. Sheffield: School of Health and Related Research (ScHARR), University of Sheffield; 2018. [cited 2026 Apr 5]. Available from: <https://view-health-screening-recommendations.service.gov.uk/bowel-cancer/#:~:text=Evidence%20summary%20Bowel%20Cancer%20(2018)>
7. US Preventive Services Task Force, Davidson KW, Barry MJ, Mangione CM, Cabana M, Caughey AB, et al. Screening for Colorectal Cancer: US Preventive Services Task Force Recommendation Statement. JAMA. 2021 May 18;325(19):1965. doi:[10.1001/jama.2021.6238](https://doi.org/10.1001/jama.2021.6238)
8. Haute Autorité de Santé. Saint-Denis La Plaine: Haute Autorité de Santé; 2013 Jan 16. [cited 2026 Apr 5]. Available from: https://www.has-sante.fr/upload/docs/application/pdf/2012-07/c_2012_0001_composition_ceesp.pdf
